# Determinants of COVID-19 Vaccination Decision-Making Behaviors among Pregnant Women in Sub-Saharan Africa: A Scoping Review

**DOI:** 10.3390/vaccines11071233

**Published:** 2023-07-12

**Authors:** Sylvia Ayieko, Kimberly Baker, Sarah E. Messiah, Brianna Lewis, Christine Markham

**Affiliations:** 1 Department of Health Promotion and Behavioral Sciences, The University of Texas Health Science Center at Houston School of Public Health, Houston, TX 77030, USA; kimberly.baker@uth.tmc.edu (K.B.); brianna.lewis@uth.tmc.edu (B.L.);; 2 Department of Epidemiology, Human Genetics and Environmental Sciences, The University of Texas Health Science Center at Houston School of Public Health, Dallas, TX 75207, USA; 3 Center for Pediatric Population Health, The University of Texas Health Science Center at Houston School of Public Health, Dallas, TX 75207, USA; 4 Department of Pediatrics, McGovern Medical School, Houston, TX 77030, USA

**Keywords:** COVID-19 vaccination, pregnant women, vaccination decision-making, Sub-Saharan Africa, vaccination acceptability/hesitancy

## Abstract

Despite the availability of the coronavirus disease 2019 (COVID-19) vaccination, uptake among pregnant women in Sub-Saharan Africa has been low. This scoping review aimed to identify and characterize determinants influencing COVID-19 vaccination decision-making behaviors among pregnant women in Sub-Saharan Africa. We searched five online databases for articles on COVID-19 vaccination among pregnant women in Sub-Saharan Africa. We identified studies published in English between March 2020 and April 2023 that assessed vaccine-specific issues, psychosocial constructs, and contextual factors associated with COVID-19 vaccination decision-making behaviors. Of the fourteen studies identified, over half (57.1%) were cross-sectional; three used qualitative research methods; and three involved multi-country participants. Most studies assessed COVID-19 vaccination acceptability and willingness. Overall, 85.7% of the publications examined knowledge, attitudes, or both as critical factors associated with COVID-19 vaccination. The prevalence of COVID-19 vaccine uptake during pregnancy was low in Sub-Saharan Africa (14.4–28%). While most current studies assess COVID-19 vaccination knowledge, research on maternal vaccination in Sub-Saharan Africa would benefit from the inclusion of theory-informed and driven studies that measure additional psychosocial factors and contextual constructs. Future studies should also employ study designs that can determine causal pathways of vaccination determinants and vaccination uptake.

## 1. Introduction 

The World Health Organization (WHO) estimated that by October 2022, over 12 million cases and 255,912 deaths related to coronavirus disease 2019 (COVID-19) would have been reported in Africa [1,2] Although COVID-19 cases in Sub-Saharan Africa account for approximately 2% of all those reported globally [1,3], most countries in Sub-Saharan Africa have inadequate epidemiological surveillance systems and have likely underestimated the impact of COVID-19 on maternal health outcomes [4,5]. Studies have consistently shown that pregnancy increases the risk of COVID-19-related complications [6,7]. In a cohort study of several sub-Saharan African countries, pregnant women infected with COVID-19 had a higher likelihood of intensive care unit (admissions (adjusted risk ratio: 2.0), respiratory support (adjusted risk ratio: 1.57), and in-hospital mortalities (adjusted sub-hazard ratio: 2.00) [8,9,10]. Data from South Africa indicated a 3.4% rise in perinatal mortality due to COVID-19 [11], while researchers in Kenya reported disruptions in maternal health services at the onset of the COVID-19 pandemic [12]. Due to this strong evidence, public health practitioners recommend vaccination to reduce adverse outcomes related to COVID-19 infections among pregnant women [13]. 

Given the high risk of complications during pregnancy, health professionals urged countries to prioritize COVID-19 vaccination [8]. Most countries in Sub-Saharan Africa joined the COVID-19 Vaccine Global Access (COVAX) partnership program with the goal of vaccinating the majority of their populations by the end of 2022 [14,15,16]. However, COVID-19 vaccination coverage has been sub-optimal in Africa, with only 37.5% of the population receiving at least one dose of the COVID-19 vaccine by 8 May 2023, compared to 70–80% in high-income countries [2,16,17]. In Sub-Saharan Africa, previous studies have reported that pregnant women are hesitant to receive the COVID-19 vaccination despite the severe complications associated with COVID-19 infections during pregnancy [8,10]. Moreover, excluding pregnant women from the initial vaccination trials resulted in skepticism about the vaccine’s safety during pregnancy [18]. However, subsequent clinical studies show that pregnant women vaccinated against COVID-19 had lower odds of ICU admission, preterm births, or adverse perinatal outcomes than unvaccinated pregnant women [19,20] suggesting COVID-19 vaccination could benefit pregnant women.

While psychosocial determinants such as attitudes, perceived risk, or social norms can impact intention to get vaccinated, studies in the general population indicate that other contextual issues such as employment mandates for vaccination [21], a lack of vaccines, or COVID-19 vaccination policies can also influence vaccination uptake [22]. In addition, inadequate and ill-equipped health facilities to properly store vaccines, low capacity of healthcare workers, ineffective communication strategies, and community disengagement have led to low COVID-19 vaccination coverage across Sub-Saharan Africa [23]. It is likely that similar psychosocial and contextual factors are also likely to influence COVID-19 vaccination among pregnant women in Sub-Saharan Africa. 

Previous systematic reviews focusing on vaccine safety and efficacy during pregnancy have mostly included studies conducted in Europe and the United States [24,25]. Few reviews have specifically included studies conducted in Sub-Saharan Africa assessing psychosocial, contextual, and vaccine-specific determinants of COVID-19 vaccination in pregnancy. To effectively understand the information available in the literature on COVID-19 vaccine hesitancy and intentions and increase uptake, it is crucial to identify studies examining vaccine-specific issues, psychosocial determinants, the contextual environment, and other factors related to COVID-19 vaccination behaviors. As such, we conducted a scoping review to identify and characterize studies on vaccine-specific, psychosocial, and contextual factors and COVID-19 vaccination behaviors among pregnant women in Sub-Saharan Africa. The review sought to identify knowledge gaps in the literature on COVID-19 vaccination during pregnancy and the application of theory-based research in Sub-Saharan Africa. The study also aimed to report factors associated with vaccination hesitancy, acceptance, intention, and uptake among pregnant women in Sub-Saharan Africa. 

## 2. Material and Methods

### 2.1. The Protocol and Registration

The scoping review protocol for this study was registered with the Open Science Framework (OSF)—https://doi.org/10.17605/OSF.IO/9MH5J on 1 March 2023. The methodology for this scoping review followed the Preferred Reporting Items for Systematic Reviews and Meta-analysis for Scoping Reviews (PRISMA-ScR) guidelines [26].

### 2.2. Eligibility Criteria

The current scoping review followed the Population, Exposure, Comparator, and Outcomes (PECO) framework [27]. The population of interest was pregnant women in Sub-Saharan Africa. The exposures were psychosocial, vaccine-specific, and contextual determinants associated with COVID-19 vaccination. The outcomes included COVID-19 vaccination acceptance, intention, or uptake. The complete inclusion and exclusion criteria are included in the supplement information, Table S1. 

We included studies published in English (observational studies, cross-sectional studies, clinical trials, case-control studies, cohort studies, and qualitative studies), government reports, studies from non-governmental organizations (NGOs), and dissertations conducted in Sub-Saharan Africa. We included studies published between March 2020 and April 2023 that addressed at least one of the exposures and at least one of the vaccination outcomes. We used the 3-year time frame because COVID-19 was declared a pandemic in March 2020 [28]. We excluded abstract-only publications, conference abstracts, reviews, commentaries, editorials, animal-model studies, studies that did not include pregnant women, studies examining only physiological aspects/clinical outcomes of COVID-19 vaccination outcomes during pregnancy, and studies conducted outside Sub-Saharan Africa. 

### 2.3. Information Sources

The literature search of published studies was conducted from the following sources: Ovid Medline, Embase, Ovid Psych INFO, African Index Medicus (AIM), and ProQuest databases. The search for sources was conducted between 5 March 2023, and 5 April 2023. Search results from each database were exported and uploaded to Rayyan software [29]. 

### 2.4. Search Strategy 

The key search terms included concepts on pregnancy, COVID-19 vaccination, and Sub-Saharan Africa using the following words and phrases: Coronavirus, COVID-19, COVID-19 vaccine, SARS-CoV-2 vaccines, “2019-nCoV Vaccine mRNA-1273”, “BNT162 Vaccine”, “Ad26COVS1”, “ChAdOx1 nCoV-19”, “coronavirus disease 2019 vaccination”, pregnancy, pregnant women, Sub-Saharan Africa, and 2020:3000. The review was time-bound, starting in March 2020. Searches on each database were conducted using similar search concepts. A final search strategy (Ovid Medline) is attached in a separate document (See Supplementary information Table S2).

### 2.5. Selection of Sources of Evidence

#### 2.5.1. Titles and Abstracts/Full-Text Screening

All citations were uploaded onto Ryann software, organized, and screened for duplicates [29]. Two reviewers (SAA) and (BL) independently conducted an initial screening of titles and abstracts for adherence to the inclusion and exclusion criteria. The two reviewers discussed disagreements and clarified the eligibility criteria. Both reviewers then evaluated the full-text screening of all identified publications for relevant publications. Discrepancies on the inclusion and exclusion criteria were resolved through consensus and discussion with other authors (CM, KB, and SM). 

#### 2.5.2. Data Charting Process

After completing the full-text screening for inclusion, a data charting tool was developed by SAA to determine variables to extract, which was verified by all authors. SAA independently charted the data from the included studies by identifying key study characteristics and noting other detailed information. The charting process was shared with BL, and data charts were updated based on findings that emerged from the data. The two reviewers discussed their findings or disagreements with the other reviewers (CM, KB, and SM) and established consensus. 

#### 2.5.3. Data Items

For each publication included in this scoping review, we abstracted data on study characteristics and organized the studies by author’s last name, publication date, country or region, study title, participants, study design, and setting. We provided an overview of each study, including the determinants influencing COVID-19 vaccination, outcomes, and conclusions. We presented findings from the review by including specific constructs reported by studies, behavioral health frameworks used, and other contextual socio-cultural aspects (politics, policies, or health systems) mentioned in the study. We also included the outcomes regarding vaccine hesitancy/acceptance, intention, and uptake rates and reported the associations with determinants. The first reviewer (SAA) initially extracted and synthesized the data using forms obtained from JBI [30] and discussed the findings with the other authors (BL, SM, KB, and CM). The authors deliberated on the studies included for review and resolved disagreements through consensus. 

### 2.6. Critical Appraisal of Individual Sources of Evidence

The first reviewer (SAA) conducted a critical appraisal for potential bias of the publications based on data collection methods and measures using the Joanna Briggs Institute critical appraisal tools [30] and the Mixed Methods Appraisal Tool (MMAT) [31]. We ranked the risk of bias as “low” if it was ≥70%, “moderate” if bias was 50–69%, and “high” if it was ≤49% [30]. The second reviewer (BL) assessed potential bias separately and compared the results. Discrepancies were resolved by discussion with their reviewer (CM). (See supplement information in Tables S3–S6). 

### 2.7. Synthesis of Results

We used a second form to group publications based on reported psychosocial factors, contextual determinants, and behavioral health theories. Due to the heterogeneity of the studies (qualitative, mixed-methods, cross-sectional, and cohort studies), we used a narrative approach to analyze the included studies by utilizing text to summarize and explain findings [32]. A meta-analysis was not feasible, given the different variables assessed in the studies.

## 3. Results

### 3.1. Selection of Sources of Evidence

A total of 748 studies were obtained from the Ovid Medline, Embase, Ovid Psych INFO, African Index Medicus (AIM), and ProQuest databases and exported to Rayyan software. After removing 116 duplicates, we screened 632 abstracts and titles. Following an initial screening, 569 studies were excluded, and two (2) could not be retrieved, leaving 61 publications for full-text screening. However, 49 publications did not meet the inclusion criteria, leaving 14 for the final review. 

**Figure 1:**
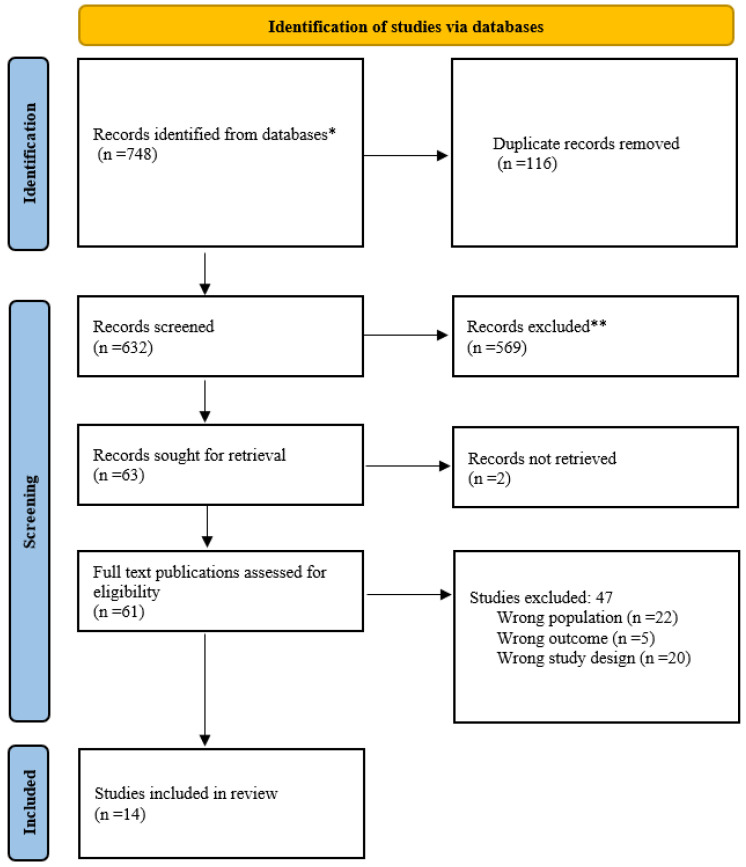
PRISMA-SCR flow diagram: Selection of sources of evidence. *—All records identified from databases; **—Records excluded after title and abstract screening.

A PRISMA-SCR flow diagram describing the selection of sources of evidence is included in Figure 1 [33]. 

### 3.2. Characteristics of Sources of Evidence

The characteristics of the included studies are described in Table 1. All the included studies were observational studies [34,35,36,37,38,39,40,41,42,43,44,45,46,47], with most (*n* = 8) incorporating a cross-sectional study design [34,36,37,38,39,40,42,45]. There was one prospective cohort study, two qualitative research studies, and three mixed-methods studies. No case-control, quasi-experimental, or randomized controlled trials were identified. The studies were conducted between 2021 and 2022. Two studies involved participants from multiple countries. The majority of the included studies were conducted in Ethiopia (*n* = 7; 50%), three in Kenya, while Cameroon and Nigeria each had one study. While ten studies were conducted in health facilities, two were conducted in the community, one was web-based, and one was conducted both online (Zoom) and in the community. Most studies included only pregnant women as study participants, although three studies compared pregnant women with other non-pregnant adults, and others included perspectives from lactating/postpartum women, healthcare workers, male family members, or policymakers. Approximately half (53%) of the quantitative studies had a sample size greater than five hundred. The study sample size in qualitative studies ranged from 18–84 participants. 

### 3.3. Critical Appraisal within Sources of Evidence

Following critical appraisals, 11 studies had a low risk of bias, while three (3) had a moderate risk. All the studies were descriptive. The qualitative studies did not provide information about the interviewers/data collectors, and there was no clarity on the research members who conducted inter-rater reliability. Two studies did not report the limitations associated with results from mixed-method designs. Many of the cross-sectional studies assessing vaccine hesitancy/acceptance reported using valid and reliable instruments. Most studies conducted appropriate statistical analysis by including multivariable regressions, but only one study identified confounding variables. The prospective study included in this review intended to measure vaccine uptake but could not adequately do so due to the unavailability of vaccines in some countries. 

### 3.4. Results of Individual Sources of Evidence

The determinants of COVID-19 vaccination during pregnancy (vaccine-specific issues, psychosocial factors, and contextual factors) are described in Table 2. Results on vaccination acceptance/hesitancy, intention, and uptake are also presented in Table 2. Table 3 shows the associations between determinants and vaccination outcomes reported in the included studies. 

### 3.5. Synthesis of Results

#### Vaccine Acceptance, Vaccine Intention, and Vaccine Uptake

Studies included in the review reported information on the variables that influenced COVID-19 vaccine decision-making processes, including vaccine acceptability (hesitancy/willingness), vaccine intention, or vaccine uptake. 

Vaccine Acceptability/Hesitancy: Eleven of the 14 studies assessed COVID-19 vaccination acceptability [34,35,36,38,40,41,42,43,44,45,46]. While other studies also assessed COVID-19 vaccination willingness and uptake, five studies examined vaccine acceptance as the only outcome. Two studies assessed vaccine hesitancy as decision-making based on the Vaccine Hesitancy Determinants Matrix [41,47]. COVID-19 vaccine acceptance was measured as a survey question or as qualitative data from interviews/focus groups. Some studies measured vaccine acceptance as Y/N in response to the question, “If you were offered a COVID-19 vaccine today, would you take it?” [38,42]. However, other studies measured vaccine acceptance by assessing willingness to receive it: “If the COVID-19 vaccine is available, are you willing to take it?” [45,46]. Overall, COVID-19 vaccine acceptance rates among pregnant women ranged from 18.5% (35) to 70.7% [42] in Ethiopia, 33.8% in Nigeria [40], and 31% in Cameroon [38]. Factors associated with vaccine acceptance/hesitancy included mistrust in health systems [34,43], perceived risk of COVID-19 infection [34,40], knowledge [35,36,42], attitudes [36,45,46], vaccine efficacy [35,40], and fear of adverse effects [43]. 

Vaccine Intention: Hailemariam et al. assessed COVID-19 vaccination intention as the only study outcome [39]. The study indicated that 31.3% of the study sample intended to receive the COVID-19 vaccination [39]. Vaccine intention was measured using a 6-point Likert scale in response to the question, “How likely do you think you are to get a COVID-19 vaccine when one is available?” Pregnant women with positive perceptions toward COVID-19 vaccines were three times (AOR: 3.04, *p* = 0.001) more likely to have COVID-19 vaccination intentions than those with negative perceptions [39]. 

Vaccine uptake: Out of the 14 studies, four studies assessed COVID-19 vaccination uptake among study participants [34,37,43,44]. The cross-sectional study conducted by Chekol Abebe et al. was the only study that examined vaccination uptake as the only outcome [37]. About 14.4% of the sample in an Ethiopian study had received the COVID-19 vaccination by March 2022, compared to 28% in Kenya [44]. Vaccination uptake was associated with higher knowledge (AOR: 3.52) and positive attitudes (AOR: 4.81) [37].

### 3.6. Vaccine-Specific Issues

Eight of the 14 studies (50%) discussed vaccine-specific issues associated with COVID-19 vaccination during pregnancy (Table 2). Vaccine effectiveness was considered among the factors influencing COVID-19 acceptance or uptake in Cameroon, Ethiopia, Kenya, and Nigeria. Study participants questioned vaccine effectiveness, with some participants stating, “I think it is not well studied” [35]. About 61.8% were uncertain about vaccine efficacy [38]. Naqvi et al. reported that fewer pregnant women in Kenya (17.4%) [43] believed that COVID-19 vaccines were very effective compared to pregnant women in the Democratic Republic of Congo (29.6%) and Zambia (48.1%) [43]. In some studies, participants questioned vaccine production reliability [38,41,47]. In Cameroon, 55.1% of pregnant participants reported that they would accept the vaccine if it were produced in Africa [38]. Since pregnant women were excluded from initial vaccine trials, some were unsure whether they were eligible for vaccines [41,47]. 

### 3.7. Psychosocial Constructs

The most common psychosocial factors assessed in the studies were knowledge/awareness (*n* = 7) and attitudes (*n* = 9). A few studies reported using validated scales to measure the various variables of knowledge and attitudes [36,42]. In most studies, participants had accurate knowledge of COVID-19 vaccines [35,36,37,42]. In a few studies, positive attitudes and knowledge were associated with vaccine acceptance [36]. However, in some studies, knowledge or attitudes were not significantly associated with COVID-19 vaccination acceptance [38,42,45].

A few studies also examined risk perceptions (*n* = 6), with mixed results on vaccine outcomes. Most studies reported a high perceived risk of COVID-19 infections, though these were not significantly associated with COVID-19 vaccination acceptance or uptake. Approximately 40% of the included studies reported fear/worry of side effects (*n* = 6), especially for the newborn, as reasons behind vaccine hesitancy or refusal during pregnancy. Tefara et al. report a participant’s concern as “I am scared that the vaccine will pass to my baby and my baby will die. My baby could not resist the side effects” [46].

Study participants’ decisions to accept vaccines were also associated with their trust in authorities (health care providers, politicians, and the government [34,38,43]. Trust was associated with vaccine hesitancy/refusal [34,43] and healthcare providers were reported as the most trusted sources of information [38]. In some cases, pregnant women did not trust the vaccine if they perceived that healthcare providers were also unvaccinated [35]. 

### 3.8. Contextual Influences 

Studies using qualitative and mixed methods explored contextual factors influencing COVID-19 vaccination among pregnant women in Sub-Saharan Africa. Salient influences included social media, myths and misinformation, and a lack of clear communication with health care providers. Amiebenomo et al. [34] reported participants’ references to historical events such as the Tuskegee syphilis study and claims of infertility in Kenya because of unverified speculations that tetanus vaccines from United Nations health agencies were adulterated [34]. However, in many studies, healthcare workers played a significant role in COVID-19 vaccination decision-making. Some myths and misinformation about COVID-19 vaccination were that the vaccine would cause infertility (contraceptive method), low libido in men, facial deformation, or would be used to “implant digital microchips” to control people’s minds. Regarding politics/policies, some studies reported a lack of policy guidance on vaccination during pregnancy [41,47].

In studies conducted earlier in the pandemic, inadequate policy information influenced patient-provider recommendations [40,42]. A few studies assessing the role of influential leaders in religious and political settings did not report any significant association with COVID-19 vaccine acceptance or willingness. However, in a few studies, participants were hesitant to receive COVID-19 vaccines based on their religion, with some believing that COVID-19 was a punishment and should be addressed through prayers [46]. Before vaccines became available, pregnant women were willing to pay for the vaccines when they became available [43]. However, the cost of transportation to vaccination sites was reported as a potential barrier [41]. 

### 3.9. Inclusion of Behavioral Health Theories/Models

Although several studies measured psychosocial constructs, very few mentioned behavioral health theory constructs to frame and support their studies. The Knowledge Attitudes and Practice (KAP) Survey Model was used in two studies, though the authors did not always explicitly mention it as the guiding framework [37,44]. Limaye and colleagues mention the socio-ecological approach in the title and abstract but do not explicitly provide an application of the framework in their study [41]. 

### 3.10. Associations between COVID-19 Vaccination Determinants and Outcomes

Knowledge, attitudes, and risk perceptions were significantly associated with COVID-19 vaccination acceptance/willingness to take vaccines. Positive attitudes towards COVID-19 vaccination were associated with vaccination intention. Studies that examined vaccine uptake reported significant associations with perceived risk, knowledge, and attitudes (Table 3). 

## 4. Discussion

### 4.1. Summary of Evidence 

This study reviewed fourteen studies on COVID-19 vaccination among pregnant women in Sub-Saharan Africa. The included studies, mainly cross-sectional observational studies, were conducted between March 2021 and April 2022, with the majority assessing COVID-19 vaccination acceptance as the primary outcome. Findings from the included studies conveyed vaccine hesitancy. WHO’s Strategic Advisory Group of Experts on Immunization defines vaccine hesitancy as “a delay in acceptance or refusal of vaccination despite the availability of vaccination services [48,49]. Two studies assessed intention, while one reported vaccine uptake as the dependent variable. 

Although emergency use of COVID-19 vaccines was made public in November 2020 [50,51], many countries in Sub-Saharan Africa did not receive vaccines until mid-2021 [3] through the COVAX initiative [14,15,16,52]. In addition, policies on COVID-19 vaccination during pregnancy varied across countries, as governments and health agencies relied on studies assessing vaccine safety and efficacy before making recommendations [53]. 

While it is unclear whether all countries in Sub-Saharan Africa have COVID-19 vaccines, recent data suggest vaccination efforts across countries [2,3,54]. Similar to prior systematic studies [25,55], pregnant participants in the included studies in this scoping review had low to moderate rates of COVID-19 vaccination acceptance. Many countries in Sub-Saharan Africa have scaled up vaccination efforts [3,53,54], with a few countries attaining their goals. Data on COVID-19 vaccination uptake during pregnancy in Sub-Saharan Africa is limited, as many countries report aggregate vaccination rates [54].

A systematic review of the COVID-19 vaccine among pregnant people in the United States reported COVID-19 vaccine intentions of 41–47.80% before vaccines became available [25]. Similar to our study findings, acceptability rates did not improve despite vaccine availability in the United States [25]. Rawal and colleagues reported the lack of provider counseling and fear of side effects as determinants of COVID-19 acceptance [25], results that closely matched our study outcomes. However, our study reported that family/friends, religious leaders, politicians, and the media influenced COVID-19 vaccination among pregnant women in Sub-Saharan Africa. These findings suggest the need to strengthen patient-provider communication and ensure the timely dissemination of accurate information in the region. Unlike the United States, the different country policies, health system infrastructure, and limited access to COVID-19 vaccines may have impacted vaccination acceptance and uptake. 

Low vaccine coverage in Sub-Saharan Africa can partly be attributed to vaccine hesitancy due to a lack of confidence in vaccine safety, low perceived susceptibility to COVID-19, misinformation about the vaccine, and distrust of governments and public health authorities [56]. Positive attitudes toward the vaccine and a higher perceived risk of COVID-19 infection are associated with lower vaccine hesitancy [57]. However, in Sub-Saharan Africa, due to the variations in the impact of COVID-19 across countries, studies report mixed findings about the perceived risk of COVID-19 infection [4]. Researchers have examined determinants such as perceived risk and fear of side effects due to COVID-19 vaccination in the general population [55,58] and used findings from clinical studies to address COVID-19 vaccination concerns.

Several studies in this review reported low vaccine acceptability due to concerns about vaccine efficacy and effectiveness, despite prior research indicating that COVID-19 vaccines are safe and effective during pregnancy [8,19,20]. Contrary to a study conducted in 2020 by the African CDC across 15 African countries where about 78% of women were willing to take the COVID-19 vaccine [14], vaccine acceptance rates were much lower among pregnant women in this review. Uncertainty about vaccine production has also been reported in other studies in Africa [14,59]. The fast-track vaccine development raised questions about vaccine efficacy [60], given that some pharmaceutical drugs can take decades before being authorized for use [61].

Governments should engage community health workers and individuals working directly with women at the grassroots to enhance vaccine acceptability among pregnant populations in Sub-Saharan countries. For example, public health officials could leverage the role of community influencers and faith leaders in communicating about vaccine safety and effectiveness in pregnancy. COVID-19 vaccine coverage could also be increased by offering accurate information to media houses (Radio, TV, and newspapers), religious leaders, and government officials. 

Over 90% of the included studies assessed knowledge or attitudes associated with COVID-19 vaccination, with a few authors using validated scales and providing a rationale for examining knowledge, attitudes, and practices associated with vaccination. Studies in this review reported high knowledge rates about COVID-19, although this was not always significantly associated with vaccine acceptance or uptake [38,45]. Positive attitudes towards COVID-19 vaccination did not always predict COVID-19 acceptance [42], which was comparable to other global studies [25]. The mixed findings from studies that examined only knowledge and attitudes as psychological factors suggest a need to address other factors that could influence vaccination. 

Some studies included in this review also examined perceived risk, trust, and fear as psychosocial constructs influencing COVID-19 vaccination decision-making or uptake during pregnancy [34,38,39]. The fear of side effects from COVID-19 vaccines could be due to limited communication about vaccine safety during pregnancy. Studies in this review reported that healthcare providers’ lack of effective communication with pregnant women has contributed to low vaccine acceptance [41,46]. Among pregnant populations in the United States, fear of adverse side effects influenced COVID-19 vaccination decision-making [25]. Some other research suggests that lack of trust in health authorities (health providers/health ministries) in Sub-Saharan Africa may be attributed to prior events, such as Polio vaccinations in Nigeria [62] and deworming efforts in Ghana [59,63], where there were concerns about the disease prevention efforts being conducted. Vaccine mistrust in Africa is linked to the history of colonial clinical and vaccine research abuse in Africa [59] and possibly the medical apartheid experienced by certain marginalized populations [64]. From the current study, it appears that unethical research, such as the Tuskegee experiment among African American populations [65], has led to mistrust in the new medical treatments [62,66] not just among African Americans but also across Black/African populations across the globe. 

This review suggests a paucity of theory-informed studies assessing COVID-19 vaccination in Sub-Saharan Africa. The Knowledge, Attitudes, and Practices (KAP) model, frequently used in public health research to explore health behaviors and related changes, has been used by several researchers to understand how knowledge of COVID-19 vaccines, attitudes towards vaccination, and COVID-19 preventative behaviors such as vaccination [67] influence vaccination outcomes (hesitancy, intentions, or uptake). Besides using the KAP model [37,44] or mentioning the socio-ecological approach [41], studies did not report using other behavioral health theories or operationalizations of constructs to understand COVID-19 vaccination determinants in pregnancy. Other COVID-19 studies in Africa have used the health belief model, the theory of reasoned action, and other behavioral theories [68,69] to understand COVID-19 vaccination among the general population. Studies that are theory-based may be helpful to address specific behaviors and tailor interventions to increase vaccine uptake during pregnancy. 

Many countries in Sub-Saharan Africa (SSA) are lower-middle-income countries (LMIC) or low-income countries (LIC) and could not afford to purchase COVID-19 vaccines for their populations until March 2021 (15). Most countries in Sub-Saharan Africa received COVID-19 vaccines under the COVAX partnership [70]. However, people became skeptical of COVID-19 vaccines after information was circulated that the “donated vaccines” were expired [71]. Lack of vaccines has also been reported in prior vaccine efforts. For example, findings from a study on influenza vaccine uptake among pregnant women in South Africa indicated that vaccine stock-outs, current illnesses, and vaccine hesitancy were associated with non-vaccination [72]. The lower rates of COVID-19 morbidities and mortalities reported in the SSA region compared to the higher magnitude of COVID-19-related deaths reported in Western countries (Europe and the Americas), China, and India [2,17] may have influenced the perceived risk of COVID-19, thus lowering the acceptability of COVID-19 vaccines. 

Similar to the results from our study, scholars investigating the uptake of other maternal vaccines, such as influenza [73] and Tetanus vaccines [74], reported lower vaccine uptake rates than recommended among pregnant women across the globe. Results from one systematic review indicated that sub-optimal influenza vaccination during pregnancy was due to low perceived risk and concerns about vaccine safety and efficacy [73]. A global study on tetanus vaccination during pregnancy also reported lower vaccination rates in lower-to-middle-income countries due to a lack of recommendations from providers [74]. Therefore, it is essential for healthcare providers to appropriately communicate with women about maternal vaccines, address concerns, and provide recommendations based on the risks. 

In this review, studies also reported that the lack of guidance on COVID-19 vaccination policies during pregnancy influenced patient-provider communication and impacted perceptions about the vaccine [41,46]. The exclusion of pregnant women during the initial COVID-19 vaccination trials contributed to concerns about the safety and effectiveness of the vaccine for both the pregnant mother and the fetus [6,18]. While subsequent cohort studies have indicated vaccine efficacy and safety [8,20,75], many countries still have restrictive policies around COVID-19 vaccination in pregnancy [53,54]. In Sub-Saharan Africa, only 15 countries have explicit policies that recommend COVID-19 vaccination for some or all pregnant people [53]. This review included studies with participants from Ethiopia, Nigeria, and Zimbabwe, where national policies permit COVID-19 vaccination during pregnancy [53], as well as participants from the Central African Republic, Democratic Republic of Congo, Equatorial Guinea, and Lesotho, where national policies do not have positions regarding COVID-19 vaccinations for pregnant people [53]. As such, the different policies may have influenced COVID-19 vaccination acceptance or uptake rates.

Future studies on maternal vaccinations should consider using cohort study designs to examine if determinants associated with vaccination acceptance during the first few months of pregnancy influence vaccination uptake towards the end of the pregnancy period. Researchers in Sub-Saharan Africa could employ design studies informed by behavioral health theories, whose results could potentially be used to design evidence-based interventions. 

### 4.2. Strengths and Limitations

A strength of the study is the focus on countries in Sub-Saharan Africa (SSA). This region has differences in country policies, capacity, and funding, which impact COVID-19 vaccination processes among pregnant women. In addition, previous COVID-19 vaccination reviews during pregnancy have often focused on clinical outcomes and demographic factors. Thus, this study provides unique perspectives on contextual and psychosocial issues not addressed by previous reviews. One limitation of this study is that countries in Sub-Saharan Africa are not only English-speaking countries. With many Francophone countries in Sub-Saharan Africa, excluding non-English publications may have restricted the sample of included studies and comprehensive perspectives on the determinants of COVID-19 vaccination in this region. In addition, factors associated with the COVID-19 pandemic have been constantly changing. Pregnant women’s opinions on COVID-19 vaccination also likely shifted based on vaccine availability, government recommendations, and scientific evidence on vaccine safety and effectiveness. COVID-19 vaccination mandates in some countries could have contributed to higher uptake rates, even in vaccine-hesitant populations. The timing of the studies probably impacted the interpretation and generalizability of the findings because some studies were conducted before vaccination was available and proven safe during pregnancy. 

## 5. Conclusions

This scoping review explored psychosocial, contextual, and vaccine-specific determinants for COVID-19 vaccination among pregnant women in Sub-Saharan Africa. Published studies suggest that pregnant women in Sub-Saharan Africa had high levels of COVID-19 knowledge but were concerned about vaccine safety and effectiveness, resulting in relatively low overall uptake, especially compared to other countries. Research on maternal COVID-19 vaccination in Sub-Saharan Africa would benefit from the inclusion of theory-informed studies that measure additional psychosocial factors and the influence of contextual issues on vaccine behaviors. With healthcare providers regarded as the most trusted source of information on COVID-19 vaccination, there is a need for timely implementation of health policies to improve vaccine acceptance. Future studies should also utilize prospective cohort or quasi-experimental design methods that connect vaccination determinants with vaccination uptake. 

## Figures and Tables

**Table 1:** Characteristics of the included studies.

Author (s), Year	Study Title	Country	Study Design	Study Setting	Participants
Amiebenomo et al., 2023 [34]	Acceptance and Risk Perception of COVID-19 Vaccination among Pregnant and Non-Pregnant Women in Sub-Saharan Africa: A Cross-Sectional Matched-Sample Study	Multi-country Cameroon * Central African Republic (CAR) Chad Democratic Republic of Congo (DRC) Equatorial Guinea Ghana * Kenya Lesotho Malawi Nigeria * South Africa * Tanzania Zambia Zimbabwe	Cross-Sectional, Matched (on pregnancy status)	Web-based	Pregnant (*n* = 54) West Africa (*n* = 30) East Africa (*n* =4) Central Africa (*n* = 8) Southern Africa (*n* = 11) Non-Pregnant Women (*n* = 77) Total: (*n* = 131)
Aynalem BY et al., 2022 [35]	COVID-19 vaccine acceptability and determinants among pregnant mothers attending antenatal care services at Debre Markos town public health institutions, Debre Markos Northwest Ethiopia: mixed study	Ethiopia	Mixed methods	Antenatal care services at Debre Markos	Pregnant mothers (*n* = 350)
Aynalem ZB et al., 2022 [36]	Factors associated with willingness to take COVID-19 vaccine among pregnant women at Gondar town, Northwest Ethiopia: A multicenter institution-based cross-sectional study	Ethiopia	Cross-sectional	Institution-based	Pregnant women (*n* = 510)
Chekol Abebe et al., 2022 [37]	COVID-19 vaccine uptake and associated factors among pregnant women attending antenatal care in Debre Tabor public health institutions: A cross-sectional study	Ethiopia	Cross-sectional	Antenatal care spaces in public health institutions	Pregnant women (*n* = 634)
Gunawardhana et al., 2022 [38]	COVID-19 vaccine acceptance and perceived risk among pregnant and non-pregnant adults in Cameroon, Africa.	Cameroon	Cross-sectional	Outpatient Hospital facilities	Pregnant women (*n* = 387) and non-pregnant adults (*n* = 448)
Hailemariam et al., 2021 [39]	Predictors of pregnant women’s intention to vaccinate against coronavirus disease 2019: A facility-based cross-sectional study in southwest Ethiopia	Ethiopia	Cross-sectional	Facility-based	Pregnant women (*n* = 412)
Iliyasu et al., 2022 [40]	COVID-19 Vaccine Acceptability Among Pregnant Women in Northern Nigeria	Nigeria	Cross-sectional	Health facility	Pregnant women (*n* = 399)
Limaye et al., 2022 [41]	A socio-ecological exploration to identify factors influencing the COVID-19 vaccine decision-making process among pregnant and lactating women: Findings from Kenya	Kenya	Qualitative	semi-private setting or via Zoom	Pregnant or lactating women (*n* = 31) healthcare workers (*n* = 20) male family members *(n* = 25), gatekeepers (*n* = 8)
Mose et al., 2021 [42]	A. COVID-19 Vaccine Acceptance and Its Associated Factors Among Pregnant Women Attending Antenatal Care Clinic in Southwest Ethiopia: Institutional-Based Cross-Sectional Study	Ethiopia	Cross-sectional	Institutional- antenatal care clinics	Pregnant women (*n* = 396)
Naqvi et al., 2022 [43]	Knowledge, attitudes, and practices of pregnant women regarding COVID-19 vaccination in pregnancy in 7 low- and middle-income countries: An observational trial from the Global Network for Women and Children’s Health Research	Multi-study Kenya Zambia Democratic Republic of the Congo (DRC)	Prospective, observational (Feb 2021–Nov 2021)	Multi-center sites (hospitals)	Pregnant women Kenya *(n* = 2133) Zambia (*n* = 2205) Democratic Republic of the Congo (DRC) (*n* = 368)
Ondieki et al., 2022 [44]	Knowledge, attitude, and practice of COVID-19 preventive measures among pregnant women in antenatal clinics in western Kenya	Kenya	Mixed methods (Survey, Focus Group Discussions, Key Informant Interviews)	Antenatal clinics	Pregnant women (*n* = 387) Hospital volunteers *(n* = 4)
Taye et al., 2022 [45]	COVID-19 vaccine acceptance and associated factors among women attending antenatal and postnatal care in Central Gondar Zone public hospitals, Northwest Ethiopia	Ethiopia	Cross-sectional	Institution-based Central Gondar Zone public hospitals	Women attending antenatal and postnatal care facilities (*n* = 527)
Tefera et al., 2022 [46]	A Mixed-Methods Study of COVID-19 Vaccine Acceptance and Its Determinants Among Pregnant Women in Northeast Ethiopia.	Ethiopia.	Mixed-methods	Institutional based	Pregnant Women (*n* = 702) Sub-sample of Qualitative participants (*n* = 18)
Zavala et al., 2022 [47]	Lack of clear national policy guidance on COVID-19 vaccines influences behaviors in pregnant and lactating women in Kenya.	Kenya	In-depth interviews (Qualitative)	Communities	Pregnant or lactating women (*n* = 29) healthcare workers (*n* = 20) policymakers (*n* = 10)

Results of individual sources of evidence. * More than 10% of the participants.

**Table 2:** COVID-19 Vaccine-Specific Issues, Psychosocial, and Contextual Determinants.

Author (s), Year	Country	Vaccine-Specific Issues	Psychosocial Constructs	Contextual Influences	Health Behavior Theory	Outcomes
Amiebenomo et al., 2023 [34]	Multi-country Cameroon * Central African Republic (CAR) Chad Democratic Republic of Congo (DRC) Equatorial Guinea Ghana * Kenya Lesotho Malawi Nigeria * South Africa * Tanzania Zambia Zimbabwe	Vaccine production Vaccine development Unavailability of vaccines	Risk perception Trust/mistrust Vaccine Safety- (Attitude) Beliefs Historical events Side effects	Media Religious leaders Political leaders Information from providers Historical misperceptions (myths)	None	COVID-19 vaccine uptake
Aynalem BY et al., 2022 [35]	Ethiopia	Effectiveness of the vaccine	Fear of side effects Knowledge Beliefs		None	COVID-19 vaccine acceptability
Aynalem ZB et al., 2022 [36]	Ethiopia		Knowledge Attitude Intention		None	COVID 19-vaccine acceptability
Chekol Abebe et al., 2022 [37]	Ethiopia		Knowledge Attitudes		None	COVID-19 vaccine uptake
Gunawardhana et al., 2022 [38]	Cameroon	Vaccine effectiveness Vaccine production	Perceived risk Perceptions Safety (Fear of side effects) Trust	Source of information (Misinformation)	None	COVID-19 vaccine acceptability
Hailemariam et al., 2021 [39]	Ethiopia		Knowledge Perceptions- (Mis)Trust, Side effects,	Government compliance with COVID-19 guidelines	None	COVID-19 vaccine intention
Iliyasu et al., 2022 [40]	Nigeria	Efficacy	Side effects, Safety Knowledge Risk perceptions	Doctor recommendation	None	COVID-19 vaccine acceptability
Limaye et al., 2022 [41]	Kenya	Vaccine availability, accessibility, and eligibility	Vaccine Safety Risk perception	Myths Interpersonal norms Religion Role of healthcare worker	Socio-ecological constructs	COVID-19 vaccine decision-making process
Mose et al., 2021 [42]	Ethiopia	Vaccines might be ineffective	Knowledge Attitudes Fear of side effects	Source of information Media (TV/Radio) Health care professionals Extension workers Friends and family	None	COVID-19 vaccine intention
Naqvi et al., 2022 [43]	Multi-country DRC Kenya Zambia	Vaccine effectiveness Eligibility	Knowledge, Attitudes Vaccine safety Trust	Willing to pay for vaccines Religious beliefs	None	COVID-19 vaccination willingness
Ondieki et al., 2022 [44]	Kenya		Knowledge, Attitudes Perceptions Side effects	Source of information (Media and social media) Politics Religion Health facility	None	COVID-19 vaccination willingness
Taye et al., 2022 [45]	Ethiopia		Knowledge Worry Attitude	Source of information Media-(TV/radio) Health care providers Family and friends- Religious	None	COVID-19 vaccine acceptability
Tefera et al., 2022 [46]	Ethiopia		Knowledge Attitude Fear of side effects Low perceived risk	Limited information,	None	COVID-19 vaccine Acceptability
Zavala et al., 2022 [47]	Kenya	Vaccine safety	Fear of vaccines	Policy guidance on vaccination No information on vaccination Unclear communication	None	COVID-19 vaccine Hesitancy

* More than 10% of the participants.

**Table 3:** Reported associations between determinants and COVID-19 vaccination outcomes among pregnant women.

Outcomes	Author/Year	Determinant/Primary Independent Variable	(OR, Adjusted OR [AOR]) [95% CI] (*p*-Value)
Vaccine acceptance (acceptability/willingness)	Aynalem BY et al., 2022 [35]	High knowledge	AOR = 4.06, [95% CI: 1.46–11.28]
		Media as an information source	OR = 1.60, [95% CI: 0.17–14.90] **
		Friends and family as an information source	OR = 1.88, [95% CI 0.18–19.68] **
	Aynalem ZB et al., 2022 [36]	High knowledge	AOR = 2.39, [95% CI: 1.14–5.00]
		Positive attitude	AOR = 2.13, [95% CI: 1.35–3.36]
	Gunawardhana et al., 2022 [38]	High knowledge	AOR = 2.26. [95% CI: 1.03–5.68] (*p* = 0.058) **
	Iliyasu et al., 2022 [40]	Low perceived risk	AOR = 1.24 [95% CI: 1.07–2.74]
		Vaccine safety	AOR = 8.30, [95% CI: 4.41–15.62]
		Vaccine efficacy	AOR = 1.41 [95% CI: 0.41–4.87] (*p* = 0.59) **
		Worry	AOR = 1.70 [95% CI: 0.43–6.77] (*p* = 0.45) **
	Mose et al., 2021 [42]	High knowledge	AOR = 5.95 [95% CI; 3.15-7.07)]
		Positive attitude	AOR = 1.72, [95% CI: 0.77–3.85] **
	Taye et al., 2022 [45]	High knowledge	AOR = 0.91, [95% CI: 0.53–1.56] **
		Positive attitude	AOR = 8.54, [95% CI: 5.18–14.08]
		Worry	AOR = 3.46, [95% CI: 2.16–5.52]
	Tefera et al., 2022 [46]	Positive attitude	AOR = 1.59, [95% CI: 1.09–2.31]
Vaccine intention	Hailemariam et al., 2021 [39]	High knowledge	AOR = 1.68, [95%CI: 0.88–3.21] (*p* = 0.115) **
		Positive attitude	AOR = 3.04, [95%CI: 1.64–5.62]
		Low perceived risk	AOR = 1.74, [95%CI: 0.86–3.52] (*p* = 0.12) **
Vaccine uptake	Amiebenomo et al., 2022 [34]	Low perceived risk	AOR = 1.58, [95% CI: 1.24–2.01]
		Misperceptions	AOR = 3.63, [95% CI: 2.12–11.79]
	Chekol Abebe et al., 2022 [37]	High knowledge	AOR = 3.52, [95%CI: 1.83–3.87]
		Positive attitude	AOR = 4.81, [95% CI: 1.42–7.33]

** Non-significant associations between a determinant and a COVID-19 vaccination behavior outcome. OR = Odds Ratios; AOR = Adjusted Odds Ratios; CI=Confidence Interval; *p*-value = probability value.

## Data Availability

No new data were created or analyzed in this study. Data sharing is not applicable to this article.

## Supplementary Materials

The following supporting information can be downloaded at: https://www.mdpi.com/article/10.3390/vaccines11071233/s1. Table S1: The complete inclusion and exclusion criteria; Table S2: Full electronic search Strategy for OVID-Medline search; Table S3: Risk of Bias Assessment using the Joanna Briggs Institute (JBI) Critical Appraisal Checklist for Cohort Studies; Table S4: Risk of Bias Assessment using the Joanna Briggs Institute (JBI) Critical Appraisal Checklist for Cross-Sectional Studies; Table S5: Risk of Bias Assessment using the Joanna Briggs Institute (JBI) Critical Appraisal Checklist for Qualitative Research; Table S6: Risk of Bias Assessment of Mixed Methods Study using MMAT criteria.
