# Supplementary material for: Determinants of COVID-19 Vaccination Decision-Making Behaviors among Pregnant Women in Sub-Saharan Africa: A Scoping Review
Source: Vaccines (Basel). 2023 Jul 12;11(7):1233. doi: 10.3390/vaccines11071233 (PMC10384512; doi:10.3390/vaccines11071233)
Supplement: Supplementary file 1 [file vaccines-11-01233-s001.zip › vaccines-2438337-supplementary.pdf]

Supplementary Materials

Table S1. The complete inclusion and exclusion criteria .

PECO	Inclusion Criteria	Exclusion criteria
Population/ Problem	Must include pregnant women, studies about COVID-19 vaccination,	Participants do not include pregnant individuals
Exposure	Studies examining vaccine issues (new vaccine, scheduling), psychological factors (attitudes, beliefs), and contextual determinants (policies, misinformation)	Studies examining clinical or biological determinants only
Context	Studies must be conducted in Sub-Saharan Africa	Studies among African women residing in other countries
Outcome	Vaccination intention, hesitancy/ acceptance, uptake	
Types of studies	Observational studies, cross-sectional studies, clinical trials, case-control studies, cohort studies, qualitative studies	Abstract-only publications, conference abstracts, reviews, commentaries, editorials
Time frame	Published between March 2020 and January 2023	

Table S2. Full electronic search Strategy for OVID-Medline search.

Concepts	Search terms
**Pregnancy**	"Pregnancy"[Mesh] OR "Pregnant Women"[Mesh]) OR "Prenatal Care"[Mesh] OR pregnan*, OR expectant* OR expecting* OR matern*
**COVID-19 vaccination**	"COVID-19 Vaccines"[Mesh] OR "COVID-19"[Mesh] OR "SARS-CoV-2"[Mesh]) OR "2019-nCoV Vaccine mRNA-1273"[Mesh] OR "ChAdOx1 nCoV-19"[Mesh] OR "BNT162 Vaccine"[Mesh]OR "Ad26COVS1"[Mesh] OR "covid-19 vaccin*" OR "Sars-cov-2 vaccin*" OR "SARS COV 2 vaccin*" OR "2019-nCoV Vaccine mRNA-1273" OR "BNT162 Vaccine" OR "Ad26COVS1" OR ("COVID 19 virus vaccin*" OR ("ChAdOx1 nCoV-19" OR "coronavirus disease 2019 vaccin*”
**Sub-Saharan Africa**	"Africa South of the Sahara"[Mesh] OR "Africa, Western"[Mesh] OR "Africa, Southern"[Mesh] OR "Africa, Eastern"[Mesh] OR "Africa, Central"[Mesh] OR "Africa"[Mesh] OR "Developing Countries"
**Language**	English
**Date of publication**	2020:3000

Truncation wildcard used to find word endings for example vaccin was used to find “vaccine”, “vaccines”, “vaccination”, or “vaccinated”

Table S3. Risk of Bias Assessment using the Joanna Briggs Institute (JBI) Critical Appraisal Checklist for Cohort Studies.

	**Naqvi et al., 2021**	**Yes**	**No**	**Unclear**
1	Were the two groups similar and recruited from the same population?	✓		
2<br>3	Were the exposures measured similarly to assign people to both exposed and unexposed groups?			??
4	Was the exposure measured in a valid and reliable way?	✓		
5	Were confounding factors identified?	✓		
6	Were strategies to deal with confounding factors stated?		X	
7	Were the groups/participants free of the outcome at the start of the study (or at the moment of exposure)?	✓		
8	Were the outcomes measured in a valid and reliable way?	✓		
9	Was the follow up time reported and sufficient to be long enough for outcomes to occur?	✓		
10	Was follow up complete, and if not, were the reasons to loss to follow up described and explored?			??
11	Were strategies to address incomplete follow up utilized?			??
12	Was appropriate statistical analysis used?	✓		
	Total	**58%**	**Moderate risk of bias**	

Note: The risk of bias was ranked as high when the study reached up to 49% of "yes" scores, moderate when the study reached from 50 to 69% of "yes" scores, and low when the study reached more than 70% of "yes" scores. '✓'indicates yes, '✕'indicates no and '?'indicates unclear.

Table S4. Risk of Bias Assessment using the Joanna Briggs Institute (JBI) Critical Appraisal Checklist for Cross-Sectional Studies.

Studies	1. Were the criteria for inclusion in the sample clearly defined?	2. Were the study subjects and the setting described in detail?	3.Was the exposure measured in a valid and reliable way?	4. Were objective, standard criteria used for measurement of the condition?	5. Were confounding factors identified?	6. Were strategies to deal with confounding factors stated?	7. Were the outcomes measured in a valid and reliable way?	8. Was appropriate statistical analysis used?	% Yes	Risk of bias
Amiebenomo, 2023	✓	✓	?	✓	?	╳	✓	✓	63%	Moderate
Aynalem ZB, 2022	✓	✓	✓	✓	?	✓	✓	✓	88%	Low
Chekol Abebe, 2022	✓	✓	✓	✓	?	?	✓	✓	75%	Low
Gunawardhana, 2022	✓	✓	✓	✓	✓	✓	✓	✓	100%	Low
Hailemariam, 2021	✓	?	✓	✓	?	✓	✓	✓	75%	Low
Iliyasu, 2022	╳	╳	✓	✓	?	✓	✓	✓	63%	Moderate
Mose, 2021	✓	✓	✓	✓	?	?	✓	✓	75%	Low
Taye, 2022	✓	✓	?	✓	?	✓	✓	✓	75%	Low

Note: The risk of bias was ranked as high when the study reached up to 49% of "yes" scores, moderate when the study reached from 50 to 69% of "yes" scores, and low when the study reached more than 70% of "yes" scores. '✓'indicates yes, '╳'indicates no and '?'indicates unclear.

Table S5. Risk of Bias Assessment using the Joanna Briggs Institute (JBI) Critical Appraisal Checklist for Qualitative Research.

	**Limaye, 2022**	**Zavala, 2022**
1. Is there congruity between the stated philosophical perspective and the research methodology?	✓	✓
**2.** Is there congruity between the research methodology and the research question or objectives?	✓	✓
**3.** Is there congruity between the research methodology and the methods used to collect data?	✓	✓
**4.** Is there congruity between the research methodology and the representation and analysis of data?	?	?
**5.** Is there congruity between the research methodology and the interpretation of results?	✓	✓
**6.** Is there a statement locating the researcher culturally or theoretically?	?	?
**7.** Is the influence of the researcher on the research, and vice- versa, addressed?	?	?
**8.** Are participants, and their voices, adequately represented?	✓	✓
**9.** Is the research ethical according to current criteria or, for recent studies, and is there evidence of ethical approval by an appropriate body?	✓	✓
**10.** Do the conclusions drawn in the research report flow from the analysis, or interpretation, of the data	✓	✓
**Yes %**	70%	70%
**Risk of Bias**	Low	Low

Note: The risk of bias was ranked as high when the study reached up to 49% of "yes" scores, moderate when the study reached from 50 to 69% of "yes" scores, and low when the study reached more than 70% of "yes" scores. '✓'indicates yes, '✕'indicates no and '?'indicates unclear.

Table S6. Risk of Bias Assessment of Mixed Methods Study using MMAT criteria.

Author, year	Are there clear qualitative and quantitative research questions (or objectives*), or a clear mixed methods question (or objective*)?	Do the collected data allow address the research question (objective)? E.g., consider whether the follow-up period is long enough for the outcome to occur (for longitudinal studies or study components).	5.1. Is the mixed methods research design relevant to address the qualitative and quantitative research questions (or objectives), or the qualitative and quantitative aspects of the mixed methods question (or objective)?	5.2. Is the integration of qualitative and quantitative data (or results*) relevant to address the research question (objective)?	5.3. Is appropriate consideration given to the limitations associated with this integration, e.g., the divergence of qualitative and quantitative data (or results*) in a triangulation design?	Score	Risk of bias
Aynalem BY, 2022	✓	✓	✓	✓	?	80%	Low
Ondieki, 2022	✓	✓	✓	?	✓	80%	Low
Tefera, 2022	✓	✓	✓	✓	?	80%	Low

Notes: *These two items are not considered as double-barreled items since in mixed methods research, (1) there may be research questions (quantitative research) or research objectives (qualitative research), and (2) data may be integrated, and/or qualitative findings and quantitative results can be integrated. The risk of bias was ranked as high when the study reached up to 49% of "yes" scores, moderate when the study reached from 50 to 69% of "yes" scores, and low when the study reached more than 70% of "yes" scores. '✓'indicates yes, '✕'indicates no and '?'indicates unclear.
